# Clinico-epidemiological Profile of Transfusion-dependent Thalassemia Patients in a Tertiary Care Children’s Hospital in Nepal: An Observational Study

**DOI:** 10.31729/jnma.v63i290.9213

**Published:** 2025-09-01

**Authors:** Bishow Nath Adhikari, Sudhir Sapkota, Sani Sipai, Biplav Ghimire, Manish Chaudhary, Hema Joshi, Arika Poudel, Amod Rayamajhi, Yoveen Kumar Yadav, Bikash Sah, Ajit Rayamajhi

**Affiliations:** 1 Department of Pediatrics, Kanti Children’s Hospital, Maharajgunj, Kathmandu, Nepal; 2 Department of Pediatrics, National Academy of Medical Sciences, Mahabaudha, Kathmandu, Nepal; 3 Department of Ophthalmology, Bajrabarahi Chapagaun Hospital, Chapagaun, Lalitpur, Nepal; 4 Department of Internal Medicine, Bassett Medical Center, Cooperstown, New York, USA; 5 Department of Public Health, Hope International College, Mahalaxmisthan, Lalitpur, Nepal; 6 Department of Pediatrics, Narayani Hospital, Birgunj, Parsa, Nepal

**Keywords:** *blood transfusion*, *epidemiology*, *pediatrics*, *thalassemia*, *Nepal*

## Abstract

**Introduction:**

Transfusion-dependent thalassemia is a major public health concern in Nepal, with limited access to comprehensive care and paucity of national data. The main objectives of this study were to explore clinico-epidemiological profile, management practices and complications among pediatric transfusion-dependent thalassemia patients at a tertiary pediatric hospital in Nepal.

**Methods:**

This is a retrospective observational study conducted at the Thalassemia Day Care Unit, Kanti Children’s Hospital, Kathmandu. Transfusion-dependent thalassemia cases registered from January 2020 to December 2024, aged less than 15 years were included. Data on demographics, clinical features, transfusion and chelation profiles, complications, and nutritional status were acquired from hospital registry and analyzed using descriptive statistics.

**Results:**

Out of 187 patients, 121 (64.71%) were males and 156 (83.42%) had β-thalassemia major. Mean age at last follow-up was 7.52±3.68 years. 83 (44.38%) were Janajatis, particularly Tharus 40 (21.39%). Median age at diagnosis was 10 months (IQR 6-22 months), with mean pre-transfusion hemoglobin level 8.86 g/dL, and average transfusion period 2.64±0.83 weeks. Among 167 (89.30%) patients receiving chelation, 37 (19.87%) had good compliance. Endocrinopathies, hepatic dysfunction and cardiac complications were observed in 104 (55.62%), 65 (34.76%) and 2 (1.07%) patients respectively. Serum ferritin levels were above 2500 ng/mL in 92 (49.19%) patients. Regular follow up was done by 56 (29.95%) patients.

**Conclusions:**

β-thalassemia major was the most common type of transfusion dependent thalassemia in our study. There was male predominance, and over half of the patients had iron overload and some form of complications of thalassemia. Regular follow up and compliance to chelation therapy has to be focused for better care of transfusion dependent thalassemia patients.

## INTRODUCTION

Transfusion-dependent thalassemia (TDT) is a severe inherited hemoglobinopathy, requiring regular blood transfusions and iron chelation. These include β-thalassemia major, hemoglobin E (HbE)/β-thalassemia and severe forms of α-thalassemia like hemoglobin H (HbH) disease.^1^ The Global Burden of Disease (GBD) study in 2021 estimated the worldwide prevalence of thalassemia cases at over 13 million, with the annual incidence of thalassemia around 120,000.^2^ Among these children born with this catastrophic blood disorder, most of them reside in low- and middle-income countries like Nepal with limited health care resources^3^

Despite the high burden, comprehensive data on TDT from Nepal are lacking. The main objective of this study was to explore the clinical and epidemiological profile, management practices, and complications of pediatric TDT patients at a tertiary pediatric hospital in Nepal. This study also aims to add to the information on TDT given by previous studies to improve understanding TDT management in Nepal.

## METHODS

This is a retrospective observational study conducted at the Thalassemia Day Care Unit of Kanti Children’s Hospital (KCH), Kathmandu, which is the national referral center with dedicated pediatric hematology unit in Nepal. The study was conducted after receiving ethical clearance from the Institutional Review Committee (IRC) of Kanti Children’s Hospital (Ref. No.1736/2081/082).

This study included all cases of TDT registered at KCH from January 2020 to December 2024. TDT cases with confirmed diagnosis of β-thalassemia major, HbE/β-thalassemia or a-thalassemia; detected by hemoglobin (Hb) electrophoresis, high performance liquid chromatography (HPLC) or genetic testing were included. Inclusion required age less than 15 years, with minimum six months of follow-up data. Children with incomplete medical records and who were diagnosed with nontransfusion-dependent thalassemia were excluded.

TDT was defined as a case of thalassemia requiring six or more red blood cell units over 6 months with ≤6-week transfusionfree period or receiving frequent transfusions for >1 year, to maintain pre-transfusion hemoglobin levels above 9.50 g/dl, as per Thalassemia International Federation (TIF) guidelines.^4^ Data were obtained from hospital registry, patient records, log books and laboratory reports, using a structured proforma. Data on demographics, clinical presentation, laboratory values, transfusion and chelation history, complications, and follow-up outcomes were included. Descriptive variables such as age, sex, ethnicity (categorized according to Central Bureau of Statistics of Nepal), and geographical distribution (based on provinces) were recorded. Anthropometric parameters were used to assess nutritional status using WHO growth standards, and classified malnutrition.^5-7^

Serum ferritin was assessed every 6 months. HIV, Hepatitis B and C serology were routinely done every 6-12 months. Oral deferasirox (20-40 mg/kg/day) or deferiprone (75-100 mg/ kg/day) were prescribed for chelation, whose drug dosages and adherence were assessed through follow-up notes and prescription records. Compliance to chelation was categorized based on self-reported intake of prescribed doses of chelators. Good compliance was defined as consumption of more than 80% of prescribed doses, fair compliance as 50-80% and poor compliance as <50% consumption.^8^ Data were entered in Microsoft Excel and analyzed using SPSS version 25.0. Descriptive statistics were described by mean, median, standard deviation (SD) and interquartile range (IQR). Categorical variables were summarized using frequencies and percentages.

## RESULTS

Among 187 patients, 121 (64.71%) were males and male-to-female ratio was approximately 1.83:1. 56 (29.95%) patients had regular follow-up while rest 131 (70.05%) had irregular hospital visits. The mean age at the last follow-up was 7.52±3.68 years. 83 (44.38%) were Janajatis, among which 40 (21.39%) were Tharus. Geographically, 61 (32.62%) patients were from Madhesh Province, followed by Bagmati 56 (29.95%) (Table 1).

**Table 1:** Socio-demographic profile of transfusion-dependent Thalassemia patients (n = 187).

Variables	n(%)
**Gender**	
Male	121(64.71)
Female	66(35.29)
**Ethnicity**	
Janajati	83(44.38)
Madhesi	41(21.93)
Brahmin/Chhetri	34(18.18)
Dalit	14(7.49)
Muslim	14(7.49)
Others	1(0.53)
**Province**	
Madhesh	61(32.62)
Bagmati	56(29.95)
Gandaki	24(12.83)
Lumbini	24(12.83)
Koshi	11(5.88)
Sudurpaschim	9(4.82)
Kamali	2(1.07)
**Consanguinity in Parents**	
Yes	2(1.07)
No	185(98.93)
**Family history of TDT**	
Yes	22(11.76)
No	165(88.24)
**Siblings affected**	
Yes	20(10.70)
No	167(89.30)

**Figure 1:**
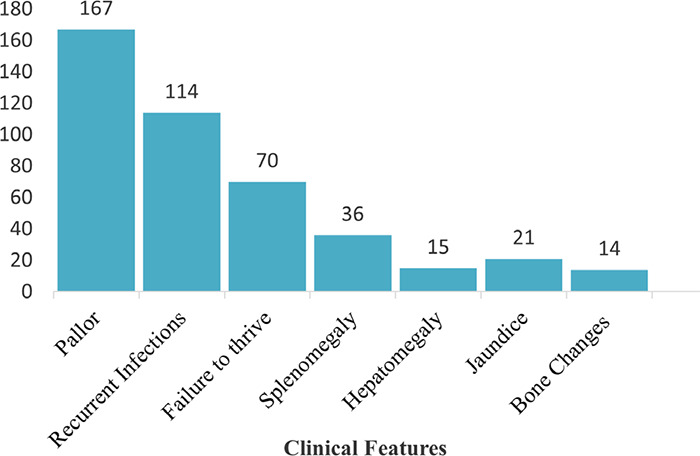
Clinical features at presentation (n=187).

Among 187 TDT patients, 167 (89.30%) patients had pallor, 114 (60.96%) patients had recurrent infections, 70 (37.43%) patients had failure to thrive while 14 (7.48%) patients had bone changes (Figure 1).

**Figure 2:**
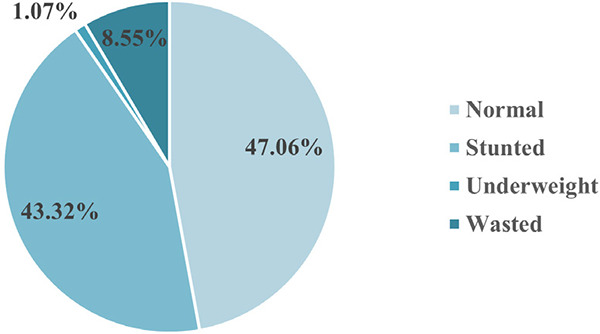
Nutritional status of transfusion-dependent Thalassemia patients (n=187).

The median age of onset of first symptom was 6 months (IQR 5-16 months) whereas the median age at the time of diagnosis was 10 months (IQR 6-22 months). There were 128 (68.45%) patients between 0-1 years of age at the time of first onset of symptoms while 15 (8.02%) were above 3 years of age. There were 104 (55.61%) patients between 0-1 years age group at the time of diagnosis while 28 (14.97%) patients were in >3-year age group at the time of diagnosis. There were 107 (57.22%) patients between 0-1 years of age at the time of first transfusion while 23 (12.30%) patients >3 years of age. Similarly, mean hemoglobin level at the time of diagnosis was 4.99±1.32 g/ dL and median age at the first transfusion was 9 months (IQR 6-21 months). There were 181 (96.79%) patients transfused packed red blood cells (PRBC) whereas 6 (3.21%) patients received mixed PRBC and whole blood. Among 187 TDT patients, there were 72 (38.50%) patients with B positive blood group followed by 54 (28.88%) patients with O positive and 41 (21.92%) patients with A positive. Mean transfusion frequency was 2.64±0.83 weeks, with an average of 22 transfusions per year. There were 10 (5.35%) patients with who had transfusion once weekly. With regard to transfusion reactions, 167 (89.30%) patients had no any transfusion reactions, 15 (8.02%) had acute febrile illness and 5 (2.67%) had allergic reaction to blood transfusion (Table 2).

Nutritional assessment showed that 88 (47.06%) patients had normal nutritional status, 81 (43.32%) patients were stunted, 2 (1.07%) underweight and 16 (8.55%) wasted (Figure 2).

Mean pre-transfusion hemoglobin threshold was 8.86±0.74 g/ dL. The median initial, peak, and recent serum ferritin levels were 804 ng/mL (IQR: 382-1342 ng/mL), 3858 ng/mL (IQR: 1951-6675 ng/mL) and 2438 ng/mL (IQR: 1092-4802 ng/mL), respectively. There were 41 (21.93%) patients with serum ferritin level <1000 ng/mL while 41 (21.93%) patients had serum ferritin level >5000 ng/mL. Among 187 patients, 71 (37.97%) had fair compliance to chelation while 59 (31.55%) patients had poor compliance to chelation. Endocrine problems were present in 104 (55.62%) of total TDT patients. Regarding endocrine problem, there were 53 (28.48%) patients with short stature only while 28 (14.97%) had both short stature and hypothyroidism. Regarding other complications, there were 65 (34.76%) patients with hepatic dysfunction, 2 (1.07%) had cardiac complication while 2 (1.07%) patients had Hepatitis C (Table 3).

**Table 2:** Clinical and transfusion profile among transfusion-dependent Thalassemia patients (n = 187).

Variable	n(%)
**Age at symptom onset (years)**	
0-1	128(68.45)
1-2	29(15.51)
2-3	15(8.02)
>3	15(8.02)
**Age at diagnosis (years)**	
0-1	104(55.61)
1-2	35(18.72)
2-3	20(10.70)
>3	28(14.97)
**Age at first transfusion (years)**	
0-1	107(57.22)
1-2	38(20.32)
2-3	19(10.16)
>3	23(12.30)
**Transfusion frequency**	
Once weekly	10(5.35)
Once every two weeks	78(41.72)
Once every three weeks	72(38.50)
Once every four weeks	24(12.83)
Once every five weeks	3(1.60)
**Age at recent follow up in years**	
0-5	57 (30.48)
5-10	77(41.18)
10-15	53(28.34)

Genetic testing was performed in 12 (6.42%) patients, identifying β-thalassemia major in 7 (3.74%) and HbH disease in 5 (2.67%) patients. Diagnosis was confirmed by Hb Electrophoresis in 170 (90.91%), HPLC in 5 (2.67%) and dual methods including genetic testing in 12 (6.42%) patients. Distribution of TDT variants showed β-thalassemia major in 156 (83.42%), HbE/β-thalassemia in 22 (11.77%), HbH disease in 5 (2.67%), and β-thalassemia intermedia in 4 (2.14%) patients.

Among 187 TDT patients, 4 (2.14%) patients had undergone splenectomy. 187 (100%) patients were on folic acid supplementation; however, none were prescribed hydroxyurea to increase fetal hemoglobin and reduce ineffective erythropoiesis.

**Table 3:** Chelation profile and complications among transfusion-dependent Thalassemia patients (n = 187).

Variables	n(%)
**Age at chelation start (years)**	
Not started	20(10.70)
<3	77(41.18)
3+	90(48.12)
**Chelation received**	
Both deferasirox and deferiprone	2(1.07)
Deferasirox	165(88.23)
Not any	20(10.70)
**Most recent ferritin (ng/ml)**	
<1000	41(21.93)
1000-2500	54(28.88)
2500-5000	51(27.26)
>5000	41(21.93)
**Compliance to chelation**	
Fair	71(37.97)
Good	37(19.78)
Not Applicable	20(10.70)
Poor	59(31.55)
**Endocrine problem**	
None	83(44.38)
Short stature	53(28.34)
Short stature and hypothyroidism	28(14.97)
Hypothyroidism	20(10.70)
Delayed puberty	2(1.07)
Hypothyroidism and delayed puberty	1(0.54)
**Other complications**	
None	118(63.10)
Hepatic dysfunction	65(34.76)
Cardiac complication	2(1.07)
Hepatitis C	2(1.07)

Bone marrow transplantation (BMT) was planned and awaited in 4 (2.14%) cases. Bone health was evaluated in all patients with mean calcium and vitamin D levels of 9.12±0.79 mg/dL and 28.29±12.36 ng/mL respectively, however bone densitometry (DEXA) scan was not done in any patients. No patients underwent magnetic resonance imaging (MRI T2*) for evaluation of iron overload. Hepatomegaly was present in 169 (90.37%) and splenomegaly in 163 (87.17%) patients.

## DISCUSSION

Thalassemia poses a significant public health challenge globally, which is evident in South Asia, including Nepal. This could be due to high carrier rates and suboptimal access to comprehensive care.^9^ Most available studies done previously in Nepal were limited to beta-thalassemia, and included few aspects of clinico-epidemiological profile and comprehensive management practices.^10-12^ Our study incorporates comprehensive profile of TDT patients including both alpha and beta variants. Genetically, thalassemia affects both genders equally, however our study shows male predominance, which aligns with other South Asian studies.^12-14^ 83 (44.38%) patients belonged to Janajati and 41 (21.93%) to Madhesi ethnic groups. These are consistent with prior reports on high carrier frequency of β-thalassemia in specific ethnic groups, predominantly Madhesi, Tharu, and Muslim populations, indicating regional clustering of hemoglobinopathies.^10,11,15^ Geographically, Madhesh Province 61 (32.62%) and Bagmati Province 56 (29.95%) had the highest representation. This may be due to a high population density, a high regional prevalence or better referral pathways.

Around 85% patients were diagnosed before three years of age, consistent with the natural history of β-thalassemia major which typically manifests with anemia during infancy or early childhood.^12,16^ Median age at diagnosis in our study was 10 months, which reflects reasonable increase in awareness among caregivers.^13^ However, delayed diagnosis beyond the first year in a notable proportion of patients remains concerning. This demands screening and preventive strategies including molecular diagnostics specific to high-risk communities such as Tharus and Muslims with high prevalence of mutations.^10,12^

Pallor 167 (89.30%), recurrent infections 114 (60.96%), and failure to thrive 70 (37.43%) were the predominant presenting complaints. These findings echo regional and global data where chronic anemia and recurrent infections are the hallmark of untreated or late-treated thalassemia.^4,13,14,16^ The average age at first symptom was 13.61 months, with diagnosis at around 17.86 months, suggesting a diagnostic delay, which was also described in other South Asian studies.^4,13^ Our study emphasizes the burden of hepatosplenomegaly, corroborating prior research, which is attributable to chronic hemolysis and extra-medullary hematopoiesis.^13,14,16^ Mean pre-transfusion hemoglobin level was 8.86 g/dL, and the recommended hemoglobin levels of 9.50-10.50 g/dL were met in a minority of patients.^3^ A significant number of our patients 96 (51.33%) were receiving regular blood transfusions at intervals of 3 to 4 weeks, with average transfusions of 22 times/year, in line with standard TDT protocols.^1,3^ Despite this, a subset of our patients had suboptimal transfusion intervals and hemoglobin levels at presentation, likely due to resource limitations, poor health knowledge, or delayed access to transfusion services, issues previously documented in Nepal and other low-resource settings.^4,11,12,13^

One of the major challenges in TDT care is iron overload due to repeated transfusions. Serum ferritin levels were elevated more than 1000 ng/mL in 146 (78.07%) patients, with 92 (49.19%) having levels > 2500 ng/mL. This is similar to findings from studies in Nepal and India, indicating a high burden of iron overload.^11,17^ Iron overload is associated with endocrine, hepatic, and cardiac complications and remains a leading cause of morbidity and mortality in inadequately managed TDT cases.^1^ In our study, despite almost 90% patients were receiving chelation therapy, 65 (34.76%) had hepatic dysfunction, and 59 (31.55%) had poor compliance. In resource-limited setting like ours, issues such as inadequate access to oral iron chelators, compounded by cost, side effects and lack of awareness about consequences of iron overload, pose a significant challenge to ensure timely and appropriate chelation therapy among TDT patients.^12,13,15,18^ These findings mandate the need for individualized chelation plans, proper counseling, and availability of multiple chelating agents including a combination therapy.^19^ Moreover, the absence of cardiac MRI T2* assessments in any of these patients, despite 2 (1.07%) patients presenting with cardiomyopathy, is concerning. This fact highlights the need to integrate cardiac monitoring into routine care, as iron-induced cardiomyopathy remains a major cause of mortality among TDT patients.^17,20^

Our study has high prevalence of endocrinopathies 104 (55.62%), predominantly short stature in 81 (43.31%) and hypothyroidism in 49 (26.20%) of total patients reflecting the long-term consequences of iron overload on the pituitary-gonadal and thyroidal axis. ^12,21^ These complications are consistent with prior studies attributing poor chelation adherence and prolonged iron overload with endocrine dysfunctions.^21^ Routine endocrine evaluation should therefore be emphasized according to TDT guidelines.^1^

Out of the total TDT patients, 4 (2.14%) had undergone splenectomy in our study. This aligns with global trends toward splenectomy avoidance due to risks of sepsis and thrombosis. However, some patients with hypersplenism may remain inadequately transfused in the absence of splenectomy.^16,22^ This mandates clear national guidelines on indications of splenectomy and post-surgical follow-up, including antibiotic prophylaxis for such TDT patients in low resource settings.^22^ hepatitis C was positive in 2 (1.07%) patients in our study which reflects improved blood safety protocols to prevent transfusion related infections in recent years. This data shows less prevalence of Hepatitis C in comparison with reports from Indian and Bangladeshi centers which may be due to difference in screening and transfusion practices.^9,23^ Despite national efforts to ensure safe blood transfusion practices, inconsistent screening across peripheral centers could contribute to the ongoing risk.^12,23^

A significant proportion of our patients have growth retardation and undernutrition, which aligns with previous regional data.^12,24,25^ This might result from suboptimal transfusion and inadequate chelation among TDT patients. Chronic anemia, iron overload, inadequate nutrition and low socioeconomic status could have contributed to this. Hence, growth monitoring and nutritional interventions need to be integrated into national thalassemia care programs.^11,13,16^

Genetic confirmation of thalassemia remains the diagnostic gold standard.^16^ However, diagnoses in our setting are mainly made on clinical grounds based on hematologic parameters, which may contribute to under or misdiagnosis in borderline or complex cases. 12 (6.42%) cases in our study underwent genetic testing. This figure reveals a critical diagnostic gap, often due to high costs, limited laboratory infrastructure, and lack of awareness.^10,12^ Curative advanced treatments such as hematopoietic stem cell transplantation (HSCT) are almost inaccessible to most families due to high costs, lack of infrastructure, and donor unavailability.^12,26,27^ This fact calls for the urgent need for establishment of pediatric HSCT centers. Emerging newer therapies such as gene therapy and luspatercept based regimens, which can be promising,^16,28^ are still not within the reach for lower-middle-income countries like Nepal.

The major limitation of our study was a retrospective study design done in a single center, thus our data may not be entirely representative ofthe whole TDT population. Absence of complete genetic profiling, growth tracking, and lack of psychosocial evaluation are other limitations. For better understanding of clinico-epidemiological profile, more complete registries and follow-up systems are needed. However, this study still reflects the real-world practice of TDT management in Nepal and highlights the need for better reporting systems in future.

## CONCLUSION

This study provides essential insights into the clinical burden and current state of TDT management at a pediatric tertiary care center in Nepal. Our study shows predominance of males among TDT patients with β-thalassemia major being the commonest type of thalassemia. Endocrine and hepatic dysfunction were the commonest complications among TDT patients in our study. Burden of malnutrition was found to be high. Despite iron overload being present among significant number of patients, compliance to chelation therapy and adherence to regular follow up was found to be suboptimal.

## Data Availability

The data are available from the corresponding author upon reasonable request
